# Models of care across settings supporting ageing in place: a narrative review

**DOI:** 10.5694/mja2.70003

**Published:** 2025-07-14

**Authors:** Maria C Inacio, Stephanie Harrison, Johannes Schwabe, Maria Crotty, Gillian E Caughey

**Affiliations:** ^1^ Registry of Senior Australians Research Centre South Australian Health and Medical Research Institute Adelaide SA; ^2^ Registry of Senior Australians Research Centre Caring Futures Institute, Flinders University Adelaide SA; ^3^ Flinders University Adelaide SA

**Keywords:** Aged, Community care, Geriatrics, Primary care, Health services for the aged, Aging

## Abstract

- Older people's preference is to age in place. With an ageing population, the demand for services that are effective in supporting older people to live at home independently has increased dramatically.
- This narrative review provides an overview of recent evidence of models of care in the aged and community care and health care sectors that contribute to supporting older people (≥ 65 years) to age in place (ie, delay or avoid entry into residential long term care).
- Overall, there is limited evidence for the identified models of care about the outcome of ageing in place, but there is evidence of positive contributions to other aspects of wellbeing.
- Complex multifactorial care models, particularly those that are person‐centred, address the health and social needs of older people in the community, include comprehensive assessment and care planning, and are delivered by a multidisciplinary clinical team, had the most consistent evidence for supporting older people to age in place.
- Specialist geriatric care and home‐based palliative team care models have robust evidence of assisting individuals to achieve their aims to stay and to die at home. However, how these complex multifactorial care models work (ie, what elements contribute to success) and how to scale up specialist team care models are substantial challenges.
- No panacea exists for supporting all people to age in place, but care integration, collaboration among care settings, and multidisciplinary person‐centred clinical care that addresses health‐related decline and challenges are consistently reported to contribute to its success.

Older people's preference is to age in place; that is, to stay at home and in their community as long as possible.^1^, ^2^ With an ageing population (16% of people aged > 65 and 2.1% aged > 85 years), and increasingly complex health and care needs, the increase in demand for health and aged care services that are effective in supporting older people to live at home independently and maintaining social connections is well documented.^2^, ^3^, ^4^, ^5^ The majority of the older population (80%) have multimorbidity, which requires ongoing care to reduce disease symptoms and burdens and delay functional and psychological consequences, alongside the biological ageing trajectory.^6^, ^7^ Models of care for the older population are based on episodic, disease‐focused, reactive and fragmented care delivery that often does not meet the heterogeneity of the older population's care needs and individual priorities.^8^, ^9^

Although formal supports to age in place are delivered through the aged care sector in Australia, multiple other care settings and providers, along with individual and societal factors, contribute to the ability to successfully age in place.^10^, ^11^, ^12^, ^13^ In addition to the complexity of contributing factors, the empirical evidence for care models and key components supporting older people to age in place is limited by a lack of studies with representative cohorts and long term follow‐up. Identification of effective models that successfully support ageing in place is required to develop policies and best practices to efficiently fulfill the increasing demands on care and social sectors in Australia. In this review, we discuss the evidence for care models across the aged and community care sector as well as critical health care sector models that can influence individuals’ ability to age in place.

This narrative review provides an overview of recent evidence of models of care in the aged and community care and health care sectors that contribute to supporting older people (≥ 65 years) to age in place. We focused on models of care that have stronger evidence of contributing to ageing in place, which has been defined here as avoiding or delaying entry into residential long term care (also known as nursing homes or care homes in other countries). We searched online databases, including PubMed (Medline), Google Scholar and the Cochrane Library, between February and March 2025, for systematic reviews, meta‐analyses, other review types, and Australian‐specific primary studies published since 2010 that focused on models of care in aged care, community care, primary care, post‐acute care and palliative care and that found a positive association with supporting older people to age in place. We also included older relevant publications identified through reviewing the identified articles’ reference lists. The search was not systematic. Examples of keywords used included “ageing in place”, “delay nursing home or aged care admission”, “avoiding nursing home or aged care admission”, “palliative care at home”, “dying at home”, “home care service”, “community care”, “community health service”, “models of care”, “care intervention”, and “multidisciplinary care intervention”. The Box provides a summary of the models of care included across care settings and their evidence to support ageing in place.

Box 1 **Summary of models of care to support ageing in place across different care settings**

**Table:** 

Models of care	Key features	Strengths and limitations	Examples in Australia
**Aged and community care models**			
Complex multifactorial care interventions	Integrated care at‐home addressing social and health needs to support independent and safe living at home Ranges from entry‐level support to high level care for individuals with complex care needs Targets multiple factors challenging older people to remain at home (eg, function, isolation, clinical care) Often federally subsidised Delivered by not‐for‐profit, for‐profit and government‐run providers	Has the most compelling evidence for avoiding or delaying entry to residential long term care^14^, ^15^, ^16^, ^17^, ^18^ Heterogeneous but person‐centred and incorporates individual preferences Supply of care services is often lower than demand; for example, wait times are 13 months for people assessed as medium priority requiring a Level 4 home care package, but people assessed as high priority are assigned approved care level within one month^2^	Australian Government, Home Care Packages Program Australian Government, Commonwealth Home Support Programme Australian Government, Support at Home Program
Transitional care or restorative care	Short term services to support functional independence after hospitalisation or to prevent general functional decline Aims to reduce further hospitalisations and delay or prevent the need for residential long term care^19^ Provided at an individual's home, in the community or residential care home Delivered by state‐funded health services, jointly funded by state and federal governments or private therapists	Internationally, evidence for these programs to reduce risk of residential long term care need is mixed^14^, ^20^, ^21^ In Australia, some evidence shows that the Transition Care Program has positive results to support older people to stay at home^22^	Australian Government, Transition Care Program Australian Government, Short‐Term Restorative Care Programme
Respite care	Support an individual and their informal carer for short periods of time Available from a few hours to a few days or longer and provided in an individual's home, in the community or in residential care Often federally subsidised	Internationally, limited evidence to support older people to stay home^16^, ^23^, ^24^, ^25^ Nationally, using residential respite as intended (ie, returning home after use) achieves the goal of helping people stay living at home^24^ Increasingly, individuals are entering permanent residential long term care directly after using residential respite (52% in residential long term care came directly from respite care in 2019–20, compared with 26% in 2010–11)^26^	Australian Government, Residential Respite Care Australian Government, Community Respite under the Commonwealth Home Support Programme including Flexible Respite, Centre‐Based Respite or Cottage Respite
Home modifications, smart home and wearable devices	Interventions to enhance an older person's independence and improve quality of life Home modifications may be available through federally funded programs	Home modifications associated with a lower risk of entry into residential long term care for those with moderate to severe frailty^27^ Smart home and wearable devices may support older people to retain independence in the community, but evidence for ageing in place is unclear^28^, ^29^	Australian Government, home modifications provided on its own through the Commonwealth Home Support Programme or as part of the Home Care Packages Program Smart devices for falls detection and prevention, health monitoring
Housing models	Community‐based arrangements that support older people to stay within communities	Have demonstrated value in improving social relations and engagement, health and wellbeing, and autonomy^30^ No clear evidence of the long term benefits of housing models in reducing the likelihood or delaying entry into residential long term care	Retirement villages, congregated housing, supported and assisted living and retirement communities
Social welfare support	Not a care model, but related to an individual's ability to access care is critical to support older people, especially those no longer employed	International evidence suggests higher income may improve access to resources that enhance an older individual's ability to age in place^31^, ^32^, ^33^	Australian Government, Age Pension and other social support (eg, Carer Payments and Allowances)
**Health care models: primary care**			
Patient‐centred medical home‐based model of care	Typically consists of general practitioner‐led care, part of a multidisciplinary team, aims to provide coordinated patient‐centred care^34^	A large body of evidence exists to support this approach in primary care for effective chronic disease management^34^ Direct evidence for its effectiveness to enable successful ageing in place derived from proxy measures^34^	Adopted by many general practices in Australia
General practitioner‐led comprehensive geriatric assessment	A multidisciplinary two‐step process, consisting of a multidomain assessment of medical, psychological, social and functional needs, and development of management plan	An Australian study reported a 10% lower likelihood of transitioning to residential long term care with delivery of components concordant with a comprehensive geriatric assessment by general practitioners^35^ Studies investigating comprehensive geriatric assessment in primary care have predominantly involved geriatricians^36^	Australian Government reimbursement of general practitioners (Medicare Benefits Schedule) for services that align with a comprehensive geriatric assessment: (i) health assessments for people aged ≥ 75 years; (ii) preparation of a chronic disease management plan; and (iii) coordinating the development of a team care arrangement
Community‐based complex multifactorial models (health provider‐led)	Targeting known or hypothesised determinants of independent living aimed to provide support for ageing in place that is largely primary care‐based (nursing and general practitioners)	Moderate evidence to support that coordinated complex multifactorial interventions that are delivered within a multidisciplinary clinical team, are centrally coordinated by primary care, and include comprehensive assessment and care planning provide significant benefits to support ageing in place^18^, ^37^	Australian Government Department of Veterans’ Affairs Community Nursing Program
**Health care models: specialist team care**			
Comprehensive geriatric assessment (geriatrician‐led)	Comprehensive assessment and delivery of multidisciplinary, person‐centred interventions that encompass both clinical and social care needs	International evidence supports comprehensive geriatric assessment associated with a 23% reduced likelihood of admission to residential long term care^16^ Barriers to successful comprehensive geriatric assessment may include bringing providers into a partnership and acceptance of preventive care^38^	Australian Government Medicare Benefits Schedule items for geriatrician assessment and management plans
Rehabilitation models	Optimising function and reducing disability following illness or in association with ageing Models often focus on specific diagnoses (eg, falls, stroke) and provide short term intense interventions after a stay in hospital or in community settings following a decline in independence Can be delivered in inpatient or outpatient settings	Rehabilitation should support older people to age in place, but much of the evidence lacks clarity across settings, and definitional confusion exists between reablement, restorative care and rehabilitation^20^ Evidence supports that inpatient rehabilitation following a hip fracture likely reduces death or admission to residential long term care, but there is uncertain evidence for these outcomes for delivery in outpatient settings^39^	State‐based rehabilitation services are available, including inpatient rehabilitation, rehabilitation in the home, day rehabilitation, outpatient rehabilitation and telerehabilitation, for many conditions, including stroke, brain injury, neurological conditions and cancer
Home‐based palliative care models	Provision of comprehensive, medical, nursing, and supportive services to people with serious life‐limiting illnesses in their own homes Home‐based palliative care delivered through outpatient models	Significant evidence generally supports home‐based palliative care as a safe and successful care model, with consistency across a wide range of outcomes, especially when delivered through in‐home, specialist, or integrated care models^40^, ^41^, ^42^, ^43^, ^44^, ^45^ Evidence for the outpatient model is sparse, but may be a good alternative when in‐home models are not viable^40^	State‐based palliative care services and general practitioner‐supported care (68% of services are delivered outside of hospital, such as in the person's home in 2022–23)^46^

## Aged and community care models

Most formal aged and community care in Australia is federally subsidised and delivered by a mix of not‐for‐profit and for‐profit providers and by government‐run organisations. A significant transition for home‐based aged care service delivery will commence in November 2025, with the Support at Home Program transitioning over a period of two years.^47^ Currently, there are two major national programs responsible for the bulk of formal home care support for older people: the Home Care Packages Program (2013–2025), which delivers ongoing long term care, and the Commonwealth Home Support Programme (2017–2025), which delivers episodic care.^48^ In 2023–24, almost 335 000 people received long term home care and 835 000 episodic home based care through these programs,^48^ both significant increases from prior years. These increases were largely driven by the recommendations made by a recent Royal Commission into Aged Care Quality and Safety (2018–21)^49^ and subsequent Australian Government Aged Care Reforms (2021 – ongoing) to address the increasing demand for home‐based services.^50^, ^51^, ^52^ In addition to these home‐based aged care services, which are complex multifactorial care models, residential respite care and other flexible care programs delivered in the community^48^ are important contributors to supporting older people to remain at home, albeit some in a smaller scale and focusing on specific challenges and populations. Similarly, there are other community‐based care models, which are not part of the federal aged care sector, that have shown promise to support older people to live at home and include smaller scale programs and social welfare.

Complex multifactorial care interventions, with the central elements of care needs assessment and multidisciplinary interventions, have the most compelling evidence for avoiding or delaying older people entering residential long term care.^14^, ^15^, ^16^, ^17^, ^18^ Even though these types of interventions are generally heterogenous, they are person‐centred and therefore incorporate individual preferences, include care for informal or unpaid carers and target the multiple factors challenging older people to remain at home (eg, function, isolation, basic clinical care). A key characteristic is that they deliver seamless integrated care addressing social and health needs. In Australia, the long term home care support provided for the general population is the Home Care Packages Program, which attempts to support older people to live at home for as long as possible, with bundled clinical, domestic and supportive services. This program has been reported to support older people to stay at home for an increasing amount of time (from a median of 17 months in 2013–14 to a median of 21 months in 2022–23);^53^ however, no systematic assessment of what care elements contribute to successful ageing in place has been undertaken to date.

Other important measures to support older people to remain at home in Australia include care models that address the challenges that older people face in regaining functional independence after a hospital admission (eg, rehabilitation or transition care models) or to prevent general functional decline (eg, restorative care, falls prevention and frailty prevention models). These care models are delivered by state‐funded health services, which are jointly funded by the states, territories and the federal government, and are delivered by private therapists in the aged care sector. When delivered under the aged care sector, these models are usually less intense but also aim to reduce the likelihood of further hospitalisations and delay or prevent the need for residential long term care.^19^ Internationally, the evidence for these programs in reducing the risk of residential long term care need is mixed, generally arising from the difficulty in defining intervention elements, terminology, and study quality.^14^, ^20^, ^21^ In Australia, an analysis of over 120 000 older individuals who participated in the Transition Care Program between 2007 and 2015 has reported positive results in supporting older people to stay at home longer, especially when it is delivered in a home‐based setting.^22^ In this population‐based observational study, more than half of the cohort were discharged to the community after completing the program and remained at home after six months. However, for those who received transition care in residential long term care (and were likely more complex cases), 63% remained in residential long term care after six months.^22^ No similar evaluation exists for the national Short‐Term Restorative Care Programme, which is available to older people living in the community who are deemed at risk of hospitalisation.

Respite care, which is generally intended to provide temporary relief to someone's carer, either because of a change of circumstances or an emergent situation, has been suggested as a model to assist older individuals to stay at home longer. Internationally, there is limited evidence of its benefits in supporting older people to stay home longer, but, nationally, the residential respite program has had positive outcomes.^16^, ^23^, ^24^, ^25^ A national analysis of over 480 000 older people who received an approval for residential respite care between 2005 and 2015 showed that using residential respite as intended (ie, returning home after use), achieves the goal of helping people to live at home longer. However, 32% of the cohort used respite once and directly entered residential long term care without returning home.^24^ The national residential respite program is used by over 80 000 people yearly, but not just for the purpose of respite, as it is also often used as a trial for longer term residential care placement.

Of note, there are several other community‐based care or support models proposed to facilitate older people staying in the community. These include home modifications, smart home and wearable devices, and housing models, some of which have become more prominent recently within federally funded programs (ie, support for home modifications) or offered as alternative solutions to support individuals in the community (ie, alternative housing models). Home modifications include the installation of ramps and grab rails to enhance an older person's independence and improve quality of life,^54^ while helping mitigate the risk of falls and poor health outcomes among those with mobility impairments.^55^ Home modifications have been associated with a lower risk of entry into long term residential care for those with moderate to severe frailty.^27^ Smart home and wearable devices may also provide opportunities to support older people to retain independence in the community, but the evidence supporting the use of these technologies to facilitate older adults to age at home is still unclear.^28^, ^29^ Further, housing models, which are types of community‐based arrangements that could support older people to stay within communities, include villages, congregated housing and various types of retirement communities, and have demonstrated value in improving social relations and engagement, health and wellbeing, and autonomy.^30^ However, as with other more innovative models, there is no clear evidence of the long term benefits of these programs in reducing one's likelihood to enter, or delaying entry into, residential long term care.

Finally, social welfare (eg, government pensions, income assistance), which is not a care model but related to one's ability to access care models, is critical to support older people, especially those who are no longer employed.^56^ In Australia, over half of individuals aged 65 years and older and 78% of those aged 85 years and older rely on government pensions or allowances as their main source of income.^56^ International evidence has shown that socio‐economic advantage, including higher income, improves access to resources that enhance an older individual's ability to age in place.^31^, ^32^, ^33^ This includes better access to health and aged care services, more robust social support networks, and financial resources for home modifications.^31^, ^32^, ^33^ In 2025, under the new Support at Home Program, older people will be required to contribute, through a means‐tested determination, to the home‐based aged care services, which will likely influence choices made for the adoption of these services. Consequently, adequate social welfare payments are, and will continue to be, critical to reducing potential inequities in older adults’ accessing essential services that support them to continue to live at home.

## Health care models

### Primary care

In Australia, primary care is provided by a number of health professionals in addition to general practitioners, including nursing and allied health practitioners. However, general practitioners are generally the first and most frequently accessed primary care service for the older population, placing them at the centre of care delivery.^5^, ^57^ Provision of accessible and high quality primary care is essential to meet the growing health care needs of the older population. These care needs can include increased frailty and functional and cognitive decline, which are ultimately the main contributors to entry to residential long term care.^4^, ^11^, ^12^, ^13^ In 2020, 95% of people over the age of 65 years saw a general practitioner, and those living in the community with long term aged care supports saw a general practitioner on average 17 times a year.^3^, ^58^ Given the high prevalence of multimorbidity, particularly in people aged over 85 years, together with increasing frailty, the health status must be maintained and potentially optimised to prevent functional and cognitive decline, which will affect their ability to stay at home.^59^ While numerous primary care models have been developed and implemented to facilitate caring for the older population with multimorbidity and complex health conditions, and are associated with improvements in disease‐specific outcomes, quality of care (eg, access, safety), and quality of life, few have evidence of a direct effect to support ageing in place.^60^

The patient‐centred medical home‐based model of care typically consists of general practitioner‐led care, coordinated within a multidisciplinary team, that aims to provide patient‐centred care that includes self‐management and patient education.^34^ Although a large body of evidence exists to support this approach in primary care for effective chronic disease management, direct evidence for its effectiveness to enable successful ageing in place can only be derived from proxy measures. For example, a systematic review reported reduced depressive episodes and hospitalisations and improved health‐related quality of life and self‐management outcomes, all of which could be hypothesised to facilitate delaying entry to residential long term care.^34^

General practitioner‐led comprehensive geriatric assessment — traditionally conducted by geriatricians — in primary care has also been promoted to improve health outcomes for the older frail population, despite varied evidence in this setting.^36^ Central to this is the provision of a comprehensive geriatric assessment, which includes a multidisciplinary (eg, nurses, social workers) two‐step process, consisting of a multidomain assessment of medical, psychological, social and functional needs, followed by the development of a management plan.^61^ An Australian study of 69 717 older people aged 75 years and older who were living in the community and received home aged care support, which included the delivery of care components concordant with a comprehensive geriatric assessment by general practitioners (ie, management plan and multidisciplinary team care assessment), reported a 10% lower likelihood of transitioning to residential long term care for those who were least frail.^35^

Similar to the aged care community care models, moderate evidence has been reported from reviews examining community‐based complex multifactorial models targeting known or hypothesised determinants of independent living aimed to support ageing in place that are largely primary care based (eg, nursing and general practitioners).^14^, ^18^, ^62^ Concordant with the primary care models described above, key components from these complex multifactorial models include comprehensive assessment and care planning, and inclusion of a multidisciplinary team. A systematic review of 13 complex multifactorial interventions, predominantly nurse‐led, that were aimed to maintain health and autonomy and prevent disability for older people living in the community, with key components such as comprehensive assessment, good communication and liaison with general practitioners and individualised care planning, found that these interventions significantly improved older people's ability to remain at home.^14^ Similarly, a recent review and meta‐analysis of complex interventions to improve independent living and quality of life for older people living in the community, with nurses as the care coordinators within a multidisciplinary primary care team that involved holistic assessment and care planning, found that these interventions were associated with a 5% increased likelihood of living at home.^62^ A 2024 systematic review and network‐meta‐analysis of 129 studies and 74 946 participants reported that the interventions most likely to sustain independence and living at home included individualised care planning that comprised medication review and regular follow‐up.^18^ Lastly, a national program, the Department of Veterans’ Affairs Community Nursing Program, is one example of a complex multifactorial intervention, delivered by qualified nurses, successfully supporting older eligible veterans to stay at home.^37^ In a 2024 study, individuals in this program remained at home a median of 28 months, compared with 14 months in the comparison group (age and gender matched home care package recipients).^37^ The success of this program suggests that coordinated complex multifactorial interventions, delivered by clinically trained individuals and centrally coordinated by primary care, can offer significant benefits in keeping older people at home longer.

### Specialist team care

#### Geriatric medicine

Geriatricians can have a pivotal role in working with teams to deliver person‐centred and integrated care that focuses on a capacity‐based approach, inclusive of the needs and priorities of older people, with an emphasis on functional abilities, preventive strategies and rehabilitation services.^63^ Principles of geriatric medicine are based on comprehensive assessment and delivery of multidisciplinary, person‐centred interventions that encompass both clinical and social care needs.^63^ A Cochrane review of in‐hospital comprehensive geriatric assessment across nine countries and 29 trials found that it increases the likelihood of remaining at home following discharge and reduces admission rates to residential long term care in the 12 months following hospitalisation compared with usual care.^64^ In addition, the benefits of comprehensive geriatric assessment to support ageing in place were further highlighted by a recent review and meta‐analysis of 36 studies, which reported that comprehensive geriatric assessment was associated with a 23% reduced likelihood of admission to residential long term care.^16^

#### Rehabilitation

Rehabilitation models of care delivered by health services, similar to transition and restorative care in the aged care setting, focus on optimising function and reducing disability that may have arisen from an illness or in association with ageing. These models often focus on specific diagnoses (eg, falls, fractures, strokes and cancer), and provide short term relatively intense interventions for older people after a stay in hospital or in community settings following a decline in independence associated with an event such as a fall or a new diagnosis (eg, Parkinson disease).^19^ Rehabilitation, which can be delivered in inpatient or outpatient settings, should help older people recover after surgery, a fall or a serious health event such as a stroke, all of which increase significantly in risk with age. Rehabilitation should support older people to age in place, but much of the evidence lacks clarity, particularly on the effect on various settings, and definitional confusion exists between reablement, restorative care and rehabilitation.^20^ A Cochrane review examining multidisciplinary rehabilitation for older people following hip fracture, which affects about 17 000 older people in Australia yearly,^65^ concluded that inpatient rehabilitation probably results in fewer cases of death or admission to residential long term care, but was uncertain regarding these outcomes after delivery in outpatient settings.^39^

#### Palliative care

Just as most people want to age at home, most also want to die at home.^66^ Internationally, palliative care models have moved from inpatient hospital to hospices and home‐based models;^40^ for example, in the United States, 23.6% of people died at home in 2003 compared with 30.7% in 2017.^67^ However, in Australia, hospital (51%) and residential long term care (30%) were still the prevailing places of death for people in 2019, with only 15% dying at home.^68^ Home‐based palliative care refers to the provision of comprehensive, medical, nursing and supportive services to people with serious life‐limiting illnesses in their own homes. Important components of home‐based palliative care models are holistic and person‐centred assessment; skilled professional care (eg, skilled multidisciplinary teams); access to medicines, care and equipment; support for patients and their families; advance care planning; integration of services; virtual and remote technology; and educational interventions for family and informal carers.^40^ Home‐based palliative care can also be delivered through outpatient models, where these services are provided through outpatient clinics.^40^ Further differences in models include the professionals involved in care delivery, with specialist models (care provided by a professional for whom palliative care is their principal and specialist role), integrated models (care coordinated across both specialists and non‐specialists), and non‐specialist models (care provided by non‐specialist health care professionals such as general practitioners and nurses).^40^

Substantial evidence generally supports home‐based palliative care as a safe and successful care model, with consistency across a wide range of outcomes, especially when delivered through in‐home, specialist, or integrated care models (versus outpatient or non‐specialists models).^40^, ^41^, ^42^, ^43^, ^44^, ^45^ The in‐home care model is associated with positive outcomes for patients, caregiver, professionals, and health systems, including achieving the preferred place of death, improved overall health care cost, symptom relief, and quality of life of both the patient and the caregiver.^69^, ^70^, ^71^ Comparatively, evidence on the outpatient model is sparse, but suggestive that it might be a good alternative when in‐home models are not viable.^40^ In addition, in‐home care provided by specialists is the model with the clearest evidence of allowing individuals to die at home without compromising symptoms,^72^ but further investigation is needed into whether generalists might be able to achieve similar outcomes.^42^

## Care model implementation challenges and evidence gaps

Although several models of care suggested to influence successful ageing in place offer benefits to older people through improving their quality of life, health and wellbeing, and/or social support, high quality evidence of these models being associated with delaying or avoiding entry to residential long term care is less clear. It is likely that improving these multiple aspects of older people's lives will contribute to their ability to remain in the community. However, over‐reliance on proxy outcomes in place of the ultimate outcome of ageing in place could deter the scrutiny of these care models in achieving this increasingly important outcome for older Australians and informing future resource allocation.

Despite limited evidence, the care models with the most consistent evidence for supporting older people to age in place are complex multifactorial care models, particularly those that are person‐centred, address the health and social needs of older people in the community, include comprehensive assessment and care planning, and are delivered by multidisciplinary clinically trained professionals. In addition, specialist geriatric care and home‐based palliative team care models have robust supporting evidence of assisting individuals achieve their aims to stay and die at home. However, how these complex multifactorial care models work (ie, what elements of these interventions contribute to helping people age in place) and how to scale up specialist team care models, such as geriatric and home‐based palliative care, in a resource (and capacity) restricted environment are significant challenges. Further, there is a clear need to improve the integration of care delivery across the care settings that older people navigate, to improve care access and patient‐centred care to better support ageing in place.^73^ Key enablers to support integrated care in Australia have been identified and include multidisciplinary team‐based care, infrastructure supports across settings, funding models to encourage best practice and desired outcomes, appropriate governance and leadership, and supporting health system research.^74^

To understand complex multifactorial care models, their definitions, governance frameworks, target cohorts and confounding challenges must be fully investigated. This could begin with examination of longstanding federally subsidised national programs that deliver care, with the goal to support older people to stay at home independently through embedding reablement and restorative care (rehabilitation) approaches. For example, the Home Care Packages Program has a substantial number of participants with extended longitudinal follow‐up, which can be leveraged to understand the characteristics of implementation of this approach (intensity, staffing, range of services) and associated success in delaying residential long term care entry. In addition, identification of specific cohorts that benefit most from the overall program, or its specific elements, as well as the geographical locations and providers that best deliver care, can lead to significant learnings about the program nationally. Similarly, challenges in how to deliver more specialist team care with a limited workforce are also important questions that could help with national planning, program redesign and targeted investments. For example, studies that evaluate expansion of scope of practices within the health care system and examine potential contributions of allied health professionals should be conducted.

No panacea exists for supporting all people to age in place. However, care integration, collaboration among settings, and adequate person‐centred clinical care that addresses health‐related decline and challenges, preferably early, are consistently reported to contribute to its success. This highlights that successful ageing in place is not the result of just one care provider (or setting) but the ongoing responsibility of all.

## Open access

Open access publishing facilitated by Flinders University, as part of the Wiley – Flinders University agreement via the Council of Australian University Librarians.

## Competing interests

Maria Inacio was formerly employed by the *MJA* as a Deputy Medical Editor, although she was no longer an employee at the time of submission of this article.

## Provenance

Commissioned; externally peer reviewed.

## Author contributions

Inacio MC: Conceptualization, investigation, methodology, resources, supervision, writing (original draft). Harrison S: Investigation, methodology, writing (original draft). Schwabe J: Investigation, methodology, writing (original draft). Crotty M: Supervision, writing (review and editing). Caughey GE: Conceptualization, investigation, methodology, supervision, writing (review and editing).
